# Glucagon-Like Peptide-1 and Diabetes

**DOI:** 10.1155/2011/901954

**Published:** 2011-12-01

**Authors:** Matteo Monami

**Affiliations:** Geriatric Medicine and Cardiology, Diabetes Section, Careggi Teaching Hospital, 50141 Florence, Italy

This special issue was focused on the role and the effects of glucagon-like peptide-1 (GLP-1) in type 2 diabetes. This gastrointestinal hormone, which is mainly secreted after meals, enhances glucose-stimulated insulin release and inhibits food intake. The response of circulating active GLP-1 concentrations after a standard meal, as well as after an oral glucose load, has been reported to be reduced in type 2 diabetic patients in comparison with healthy subjects. Available data suggest that GLP-1 plays a relevant role in the regulation of postprandial glucose metabolism in physiologic conditions. Furthermore, the impairment of GLP-1 secretion after meals could contribute to the pathogenesis of hyperglycemia in type 2 diabetes. Several new drugs act through the GLP-1 signaling system to stimulate insulin release and regulate blood glucose levels in patients with diabetes.

This special issue includes 8 articles: three mechanistic studies and five reviews and meta-analyses, exploring the putative favourable effects of the incretins on beta-cell function and mass and on gastrointestinal motor function. The reviews and meta-analyses are focused on the promising beneficial extraglycaemic effects of the incretin-based therapy, including those on central nervous system (cognitive impairment) and cardiovascular risk. Last but not least, an interesting and intriguing review on gene therapy using expression vectors of GLP-1 and other incretin mimetics in the salivary gland for the treatment of type 2 diabetes mellitus (T2 DM) is presented.

*Matteo Monami* *Matteo Monami*

